# Adsorption behaviour of tetrabromobisphenol A on sediments in Weihe River Basin in Northwest China

**DOI:** 10.1007/s11356-022-22259-7

**Published:** 2022-08-24

**Authors:** Weihan Qiao, Xiaoyu Yuan, Luyu Dong, Yujin Xia, Xueli Wang

**Affiliations:** 1 Key Laboratory of Subsurface Hydrology and Ecological Effects in Arid Region, Ministry of Education, Chang’an University, Xi’an 710064, China; 2 School of Water and Environment, Chang’ an University, Xi’an 710064, China; 3 Zhongsheng Environmental Technology Development Co, Ltd, Xi’an 710065, China

**Keywords:** Tetrabromobisphenol A, Sediment, Adsorption

## Abstract

Tetrabromobisphenol A (TBBPA) is adsorbed on sediments in river environments, and various environmental factors have distinct effects on its adsorption behaviour. Investigating the adsorption behaviour of TBBPA on the sediments in Weihe River Basin is critical for protecting the water environment and providing a theoretical basis for the prevention and control of brominated flame retardant pollution. In this study, the adsorption behaviour of TBBPA on Weihe River sediment was investigated by conducting batch equilibrium experiments, and the effects of pH, dissolved organic matter, and ionic strength on the adsorption of TBBPA were discussed. The obtained results revealed that rapid adsorption was the main mechanism of the TBBPA kinetic adsorption process. The isothermal adsorption behaviour of TBBPA was well fitted by Freundlich model (R^2^ 99.21%) than Langmuir model (R^2^ 98.59%). The adsorption capacity for TBBPA is 34.13 mg/kg. The thermodynamic results revealed that the adsorption process of TBBPA by the sediment was a spontaneous endothermic reaction. The increase in pH and ionic strength inhibited the adsorption of sediments on TBBPA. With the increase in the humic acid concentration, the adsorption of TBBPA initially increased and subsequently decreased. Synchrotron radiation-Fourier transform infrared spectroscopy indicated that the adsorption mechanism of TBBPA on the surface of sediment was mainly π–π and hydrogen bonds. The obtained results are useful for understanding of TBBPA migration and transformation in river water bodies.

## Introduction

Tetrabromobisphenol A (TBBPA) is widely used as a brominated flame retardant in electronics, building materials, and textiles (Liu et al. 2016; George and Haggblom 2008; Bredhult et al. 2007; Zhang 2019). TBBPA can be released into the environment during product production, use, and disposal. TBBPA exhibits strong migration and accumulation characteristics and is a persistent organic pollutant (Tan et al. 2018; Wei et al. 2014). Although TBBPA can be combined into a polymer with covalent bonds, the unbound part is easily released into the ecological environment (Wadden et al. 1986). For example, TBBPA ingestion causes toxic effects in animals and plants at various trophic levels (Kitamura et al. 2002; Lilienthal et al. 2008; Chen, 2016; Nakagawa et al. 2007) and poses a health hazard to humans, animals, and plants. Cell experiment studies have revealed that TBBPA is acutely toxic, immunotoxic, neurotoxic, nephrotoxic, and hepatotoxic to animals, plants, and microorganisms (Lu et al. 2021).

TBBPA can enter rivers through dry and wet sedimentation or surface runoff. TBBPA can be absorbed in river sediments and may be subsequently desorbed to the overlying water under suitable environmental conditions. Aquatic plants and animals at various trophic levels may ingest TBBPA through exposure pathways, such as respiration and feeding of aquatic organisms. TBBPA ingestion may prove toxic. Therefore, investigating the distribution and desorption mechanism of TBBPA between water–sediment phases plays a critical role in understanding its migration and transformation patterns and ecological risks in the water environment.

Based on above reasons, the objective of this study was to investigate the sorption of TBBPA on sediment from Weihe River basin. The sorption isotherms, mechanisms, and thermodynamics of TBBPA on sediment were investigated. The effects of changing the contact time, humic acid (HA) concentration, ionic strength, pH, and temperature on TBBPA sorption were assessed. More importantly, synchrotron radiation Fourier-transform infrared spectra (SR-FTIR) were used to characterise the adsorption mechanism. The obtained results will improve our understanding of the behaviours of TBBPA in sediment and will benefit assessments of the risks posed by TBBPAs in the environment and models of the fates of TBBPA in the environment. At the same time, it provides certain theoretical support for the treatment of TBBPA pollution in rivers.

## Materials and methods

### Materials and instruments

#### Material

TBBPA standard stock solution (AccuStandard, USA) with a concentration of 10 µg/mL; ^13^C12-TBBPA standard stock solution (Wellington, USA) with a concentration of 10 µg·mL^−1^; ultrapure water was used for solution preparation and drenching.

#### Instruments

Liquid chromatography-triple quadrupole mass spectrometer (Agilent, USA), solid phase extraction instrument (Supelco, USA), C18 solid phase extraction column (Supelco, USA), constant temperature water bath oscillator (HSH-H type)), total organic carbon analyser (Elementar vario EL cube), ultrasonic cleaner (KQ-300DE type), synchrotron radiation-Fourier transform infrared spectrometer (Bruker Vertex 70v type) were used in the experiment.

### Test sediment

**Fig. 1:**
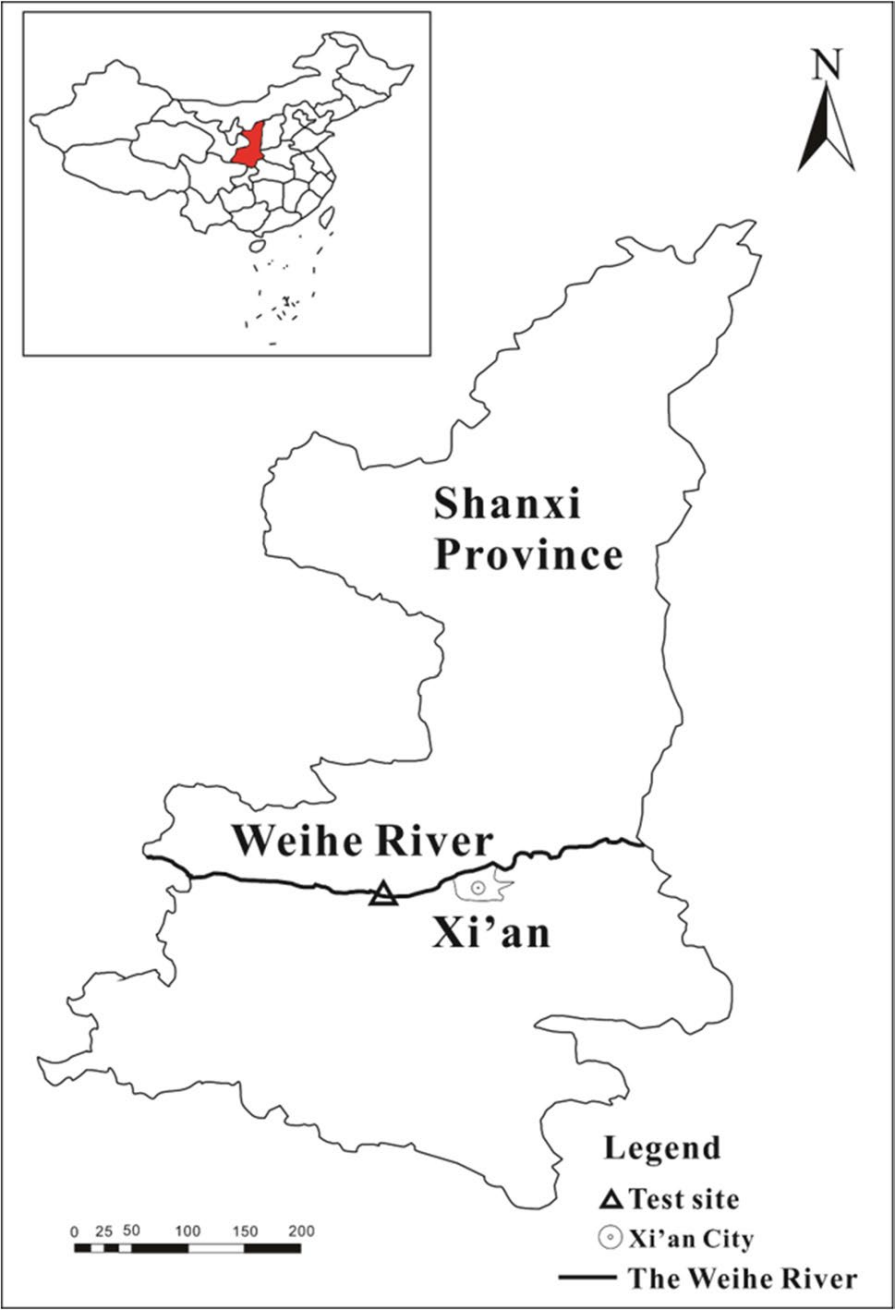
The map of Weihe River and sediment sampling site (Chen 2016)

The sediment samples were collected from the Xianyang section of the Weihe River (Fig. 1). The collected sediment samples were sealed and transported back to the laboratory, dried under natural conditions, removed from debris, ground, passed through a 60-mesh sieve, sealed, stored, and set aside.

### Adsorption experiment method

#### Adsorption kinetics experiment

The initial concentration of the target contaminant determined was 500 ng/mL by weighing several samples of 0.8 g TBBPA suspension in a 30-mL glass centrifuge tube and adding 30 mL of background solution (100 mg/L NaN_3_, 0.01 mol/L CaCl_2_) for a certain time, and then centrifuged at 15,000 r/min for 15 min. The supernatant was aspirated for analysis. During the experiment, the solvent volatilisation as well as the adsorption of the sample bottle was negligible.

The adsorption amount *Q*_t_ (ng/g) of TPPBA at moment *t* is calculated by the following equation:

1 $$\mathrm{Q}=\frac{\mathrm{V}({\mathrm{C}}_{0}-{\mathrm{C}}_{\mathrm{t}})}{\mathrm{m}}$$

where *V* (mL) is the volume of the solution, *C*_0_ (ng/mL) is the initial concentration of TBBPA, *C*_t_ (ng/mL) is the concentration at moment *t*, and *m* (g) is the mass of the suspended solids.

#### Adsorption thermodynamics experiment

Experiments were performed at 25 °C (298 K, *pH* = 7.0), 35 °C (308 K, *pH* = 7.0), and 45 °C (318 K, *pH* = 7.0) to investigate the effect of temperature variation on sediment adsorption of TBBPA. The samples were sealed and shaken at 200 rpm for a certain period of time at 25 °C. After reaching the corresponding shaking time, the samples were removed and centrifuged, and the supernatant was aspirated for analysis.

#### Experiment on the influence of environmental factors

The experiments were conducted to study the effect of pH on the sediment adsorption of TBBPA by adjusting the pH of the system to 4, 5, 6, 7, 8, 9, and 10 with 0.1 mol/L HCl and 0.1 mol/L NaOH. The ionic strength gradient was adjusted by adding the NaHCO_3_ solution at 1, 5, 10, 15, 20, 25, and 30 mg/L to study the effect of changing the ionic strength of the solution on the sediment adsorption of TBBPA. Next, the gradient was adjusted to 1, 2, 3, 5, 10, 15, 20, 25, and 30 mg/L by adding a humic acid solution to the test tube to study the effect of varying the HA concentration on the sediment adsorption of TBBPA.

#### TBBPA analysis method

TBBPA was determined through liquid chromatography-triple quadrupole tandem mass spectrometry (LC–MS/MS). For chromatographic conditions, a C18 liquid chromatographic column (150 mm × 2.1 mm, 3.0 µm, Dalian Sino-Spectrum Technology Co., Ltd.) was used for the separation, with a column temperature of 40 ℃; the mobile phase flow rate was 250 µL·min^−1^; the injection volume was 10 µL; the mobile phase gradient elution procedure is displayed in Table 1.

**Table 1:** Gradient elution programmes

*t*/(min)	Acetonitrile/%	Methanol/%	Water/%
0	55	20S	25
12	70	20	10
12.2	100	0	0
20	100	0	0
20.2	55	20	25
29.5	55	20	25

#### Mass spectrometry conditions

An ESI source and a negative ion mode were used. Detection was performed in the SIM mode. Electrospray voltage of 3000 V, capillary temperature of 230 °C, sheath gas temperature of 310 °C, sheath gas pressure of 28 psi, auxiliary air pressure of 5 psi, m/z of quantitative TBBPA ions of 542.8, and 13C12-TBBPA ions of 554.8 were used. The scan time was 250 ms, and the tube lens voltage was 75 V.

## Results and discussion

### Adsorption kinetic curve

**Fig. 2:**
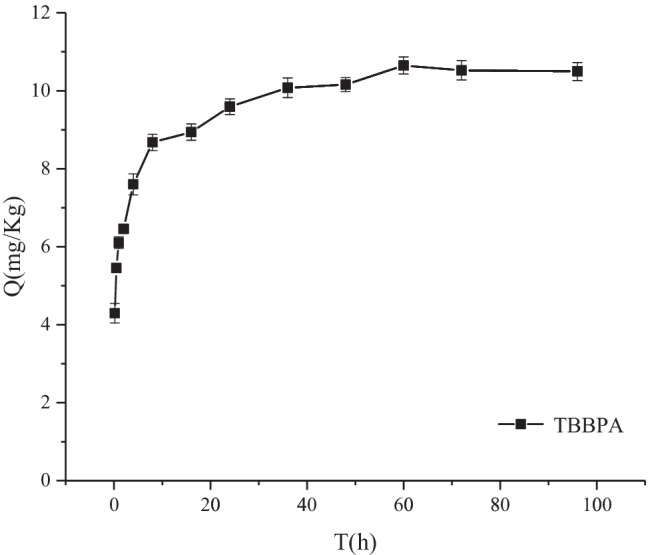
Sorption equilibrium time of tetrabromobisphenol A (TBBPA) in the sediment

The kinetic curves of sediment adsorption on TBBPA are displayed in Fig. 2. The adsorption of sediment on TBBPA in the first 2 h was a fast adsorption process, and the amount of TBBPA adsorbed reached 61.4% of the maximum adsorption amount at 2 h. After the fast adsorption step, desorption started to play a crucial role. Thus, the amount of adsorbed TBBPA changed slowly, and the adsorption reached apparent equilibrium at approximately 48 h. This result revealed that fast adsorption plays the key role in the adsorption process. The fast adsorption of TBBPA may be attributed to the adsorption of solutes on the surface of the sediment mineral medium (Huang et al. 1996) or to the distribution to the sediment organic matter (SOM) solute region (Weber and Huang 1996), whereas the slow adsorption is attributed to the gradual diffusion of TBBPA into the SOM matrix and sediment micropores (Pignatello and Xing 1996; Sun et al. 2008). Based on the results, the adsorption time was selected as 48-h adsorption and desorption in all samples for the equilibrium time experiment.

### Isothermal adsorption results

**Fig. 3:**
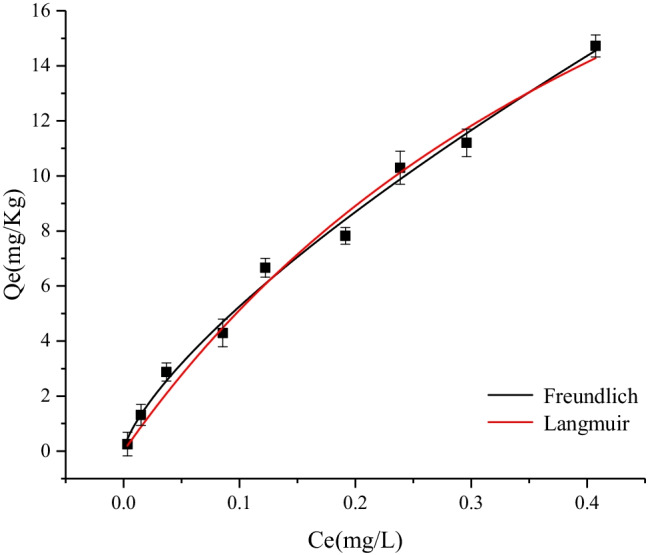
Sorption isotherms of HBCDs in the sediment

The Langmuir and Freundlich adsorption models were used to analyse the isotherm adsorption characteristics of TBBPA in Weihe River sediments. The fitting diagram is displayed in Fig. 3, and the fitting parameters are listed in Table 2.

**Table 2:** Isotherm parameters for TBBPA sorption on sediment

		Langmuir model fitting paramSeters					Freundlich model fitting parameters			
TBBPA	Q_m_ (*mg*·kg^−1^)	*K*_*L*_	*R*^2^	RSS/dof	*R*_*L*_	*K*_*F*_		*n*	*R*^2^	RSS/dof
	34.1309	1.7665	0.9859	2.3414	0.5310	27.9090		0.7249	0.9921	0.1878

Langmuir model can be expressed as follows:

2 $${Q}_{e}={Q}_{m}{K}_{L}{C}_{e}/(1+{K}_{L}{C}_{e})$$

where *Q*_e_ is the equilibrium adsorption capacity of the flame retardant in the sediment (mg/kg); *C*_e_ is the equilibrium concentration of the flame retardant (mg/L); *K*_L_ is the Langmuir coefficient; and *Q*_m_ is the maximum adsorption capacity (mg/kg). The adsorption nature of the Langmuir model can be explained by the dimensionless constant RL. When *R*_*L*_ = 0, the adsorption is irreversible; when 0 < *R*_*L*_ < 1, the adsorption can proceed; when *R*_*L*_ = 1, the adsorption conforms to linear adsorption; when *R*_*L*_ > 1, adsorption cannot be performed (Liu et al. 2010).

Freundlich model is expressed as follows:

3 $${Q}_{e}={K}_{f}{C}_{e}^{n}$$

where *Q*_e_ is the equilibrium adsorption capacity of the flame retardant in the sediment (mg/kg); *C*_e_ is the equilibrium concentration of the flame retardant (mg/L); *K*_f_ is the adsorption equilibrium constant; and *n* is the nonlinear index. The size of *n* can represent the adsorption strength of the adsorbent; that is, the larger the value of n is, the more difficult it is to adsorb, and the smaller the value of *n* is, the easier it is to adsorb.

Table 2 lists the simulation results and parameters of the adsorption isotherm curve. The correlation coefficients R^2^ of the two adsorption isotherm models are both greater than 0.9, which indicates that both the Langmuir model and Freundlich models can perform an excellent mathematical fit, but the R^2^ of the Freundlich model is greater. The residuals are smaller, so the Freundlich model fits better in comparison. The RL value of TBBPA in the Langmuir model is 0.5310, which indicates that TBBPA can be adsorbed by sediments. In the Freundlich model, *n* is 0.7249. Weihe sediments exhibit a strong adsorption capacity for TBBPA, but the K_F_ value is only 27.909 (mg/kg)/(µg/kg), which is compared with similar sediments for HBCD. The adsorption capacity is small (Wang et al. 2020). Figure 3 reveals that as the initial concentration of TBBPA increases from 0.01 to 0.4 mg/L, the equilibrium adsorption concentration increases from 0.252 to 14.7 mg/L.

### Adsorption thermodynamics results

**Fig. 4:**
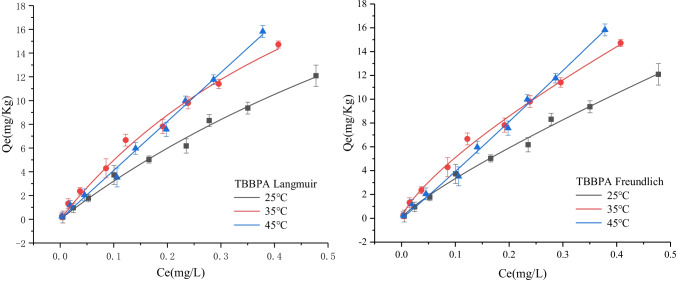
The fit of Langmuir and Freundlich model for TBBPA adsorption in sediments at different temperatures

The adsorption simulations of sediment on TBBPA were performed at 25, 35, and 45 °C, and the results are displayed in Fig. 4. Both models fit well (R^2^ is greater than 0.9), and the value of the adsorption coefficient K increases with the increase in the temperature, which indicates that the adsorption of sediment on TBBPA increases gradually with the increase of the temperature. This result could be attributed to the activation of some adsorption sites on the adsorbent surface because of the increase in the temperature, which render it easy for TBBPA molecules to enter the microporous structure.

The thermodynamic parameter entropy change (Δ*S*), enthalpy change (Δ*H*), and Gibbs free energy (Δ*G*) were calculated using Eqs. 4 and 5 (Chun et al. 2016), and the results are listed in Table 3. The Gibbs free energy (Δ*G*) of TBBPA adsorption by sediment at various temperatures was all negative, which indicate that the adsorption of TBBPA by sediment is a spontaneous process. The enthalpy changes (Δ*H*) of sediment adsorption of TBBPA were all greater than 0, which indicate that the adsorption process is a heat-absorbing reaction. Thus, increasing the temperature facilitates the adsorption. The entropy changes (Δ*S*) of sediment adsorption of TBBPA were all greater than 0, which indicated an increase in the confusion at the solid–liquid interface during the adsorption process (Li et al. 2005).

**Table 3:** Thermodynamic parameters of TBBPA adsorption on sediments

	*T* (*K*)	*K*	Δ*G* (kJ·mol^−1^)	ΔH (kJ·mol^−1^)	Δ*S* (kJ·mol^−1^)
TBBPA	298	22.35	− 7.70		
	308	28.61	− 8.31	17.11	0.083
	318	43.67	− 9.36		

4 $$\Delta \mathrm{G}=-\mathrm{RTlnK}$$

5 $$\Delta \mathrm{G}=\Delta \mathrm{H}-\mathrm{T}\Delta \mathrm{S}$$

where *R* is 8.314 J/mol·K, the ideal gas constant; *K* is the adsorption equilibrium constant; and *T* (*K*) is the absolute temperature.

### Analysis of influencing factors

#### pH

**Fig. 5:**
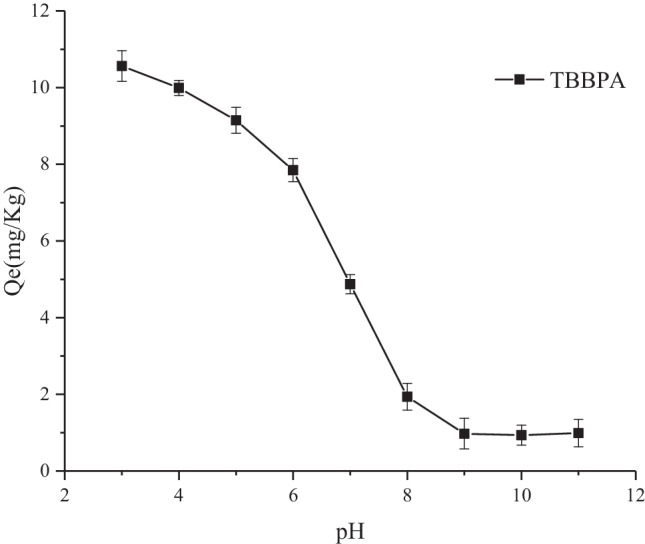
Effects of pH on TBBPA sorption in the sediment

TBBPA, as an ionisable organic compound, exhibits a charge that varies with pH. Increasing the pH reduces the adsorption performance of the sediment on ionisable organic compounds (Li et al. 2007). The effect of pH on TBBPA adsorption was studied by varying the pH of the solution, and the results are displayed in Fig. 5. The adsorption of TBBPA by the sediment decreases with the increase in pH, reaching a plateau at pH 8.0. Next, the adsorption did not change considerably with the increase in pH. This result could be because when the pH is low, TBBPA exists mainly in the form of molecules. Thus, hydrophobic interaction is the main mechanism of the adsorption process. As the pH increases, TBBPA exists in the form of anions, and the electrostatic repulsion between the negative charges on the sediment surface becomes more pronounced, and the adsorption decreases (Han et al. 2013). Furthermore, an increase in pH promotes the dissolution of organic matter in the sediment and subsequently decreases the adsorption of TBBPA.

#### Ionic strength

**Fig. 6:**
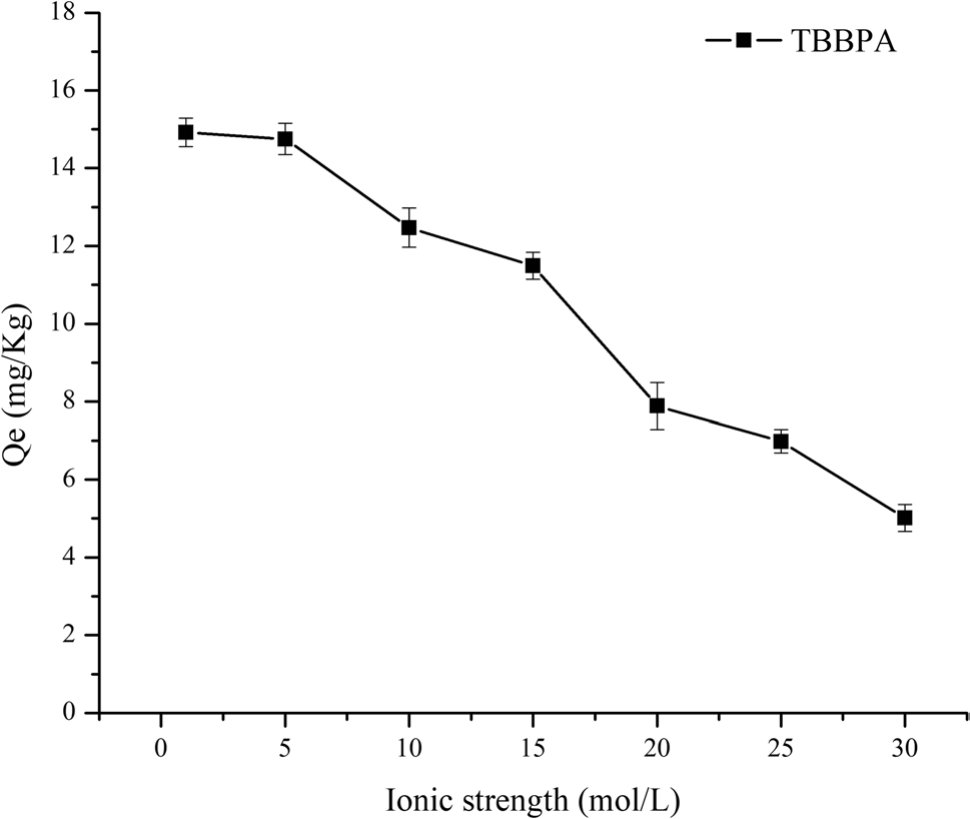
Effects of ion strength on TBBPA sorption in the sediment

The adsorption process exhibits a potential to decrease, increase, or remain constant with the increase in the ionic strength, and changes in ionic strength also alter the results of adsorption kinetics (Ye et al. 2008). In this experiment, NaHCO_3_ was used as the electrolyte to study the effect of the ionic strength on the adsorption of TBBPA by sediment. The results are displayed in Fig. 6; the adsorption of TBBPA by sediment decreases with the increase in the ionic strength, which could be because the presence of HCO_3_^−^ increases the pH in the system. Thus, pH is the main factor for adsorption and inhibition of the adsorption of TBBPA by sediment.

#### Humus (HA)

**Fig. 7:**
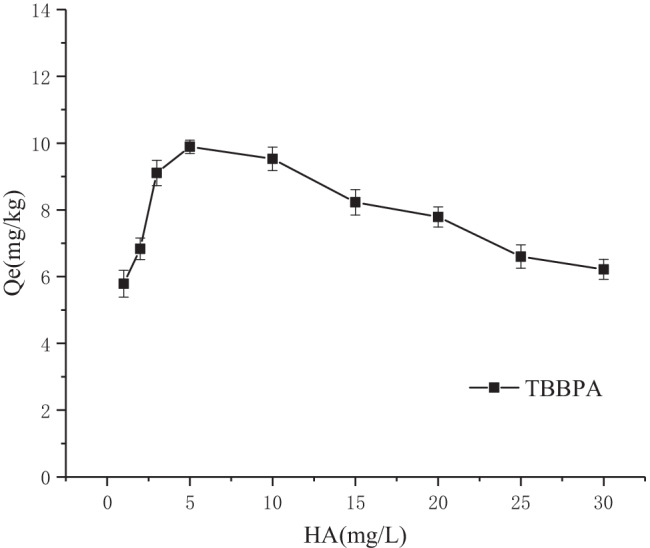
Effects of HA on TBBPA sorption in the sediment

Dissolved organic matter in river water is mainly humic acids, carbohydrates, and proteins, which are commonly found in sediments, soils, and water bodies (Tipping et al. 1999), and dissolved organic matter is a crucial and extremely active component Mcdowell 2003), which considerably influences migration, transformation, and final fate of organic pollutants in rivers. The dissolved organic matter used in this experiment was HA, and the effect of various concentrations of HA on sediment adsorption of TBBPA was investigated. The results are displayed in Fig. 7, where the sediment adsorption of TBBPA revealed a trend of increasing and subsequently decreasing with the increase in the HA concentration. This result could be attributed to the fact that in a certain range, the presence of HA forms a complex of TBBPA bound to HA, and this form is easier to be adsorbed into the sediment. When the concentration of HA exceeds 5 mg**/**L, the higher pH and excellent buffering properties increase the pH of the system, rendering the pH the dominant factor. Thus, the adsorption of TBBPA decreases by the sediment.

#### Adsorption mechanism

**Fig. 8:**
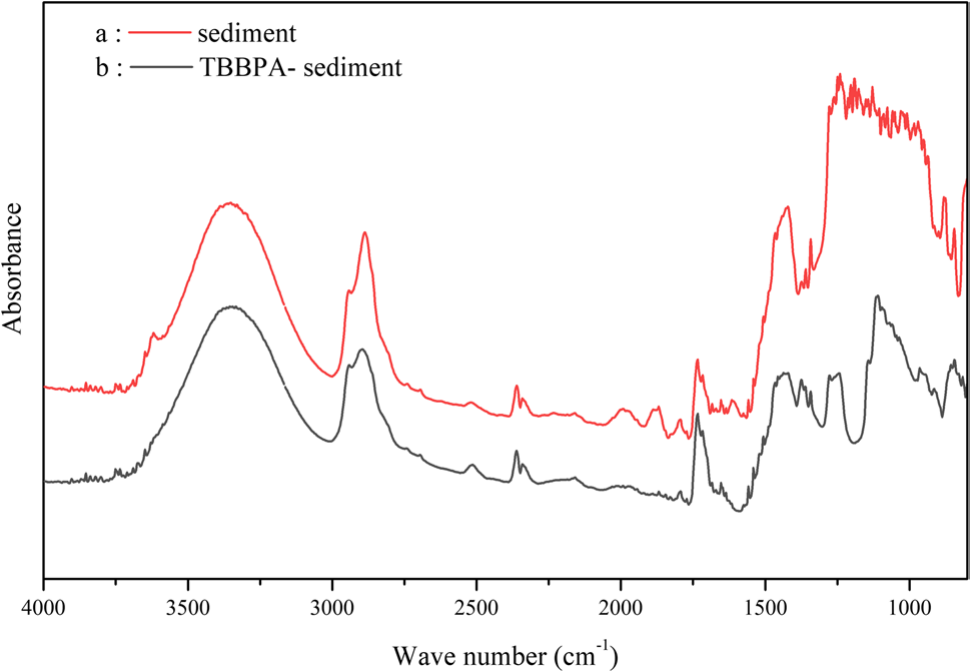
SR-FTIR spectra of **a** sediment and **b** TBBPA-sediment

The samples before and after sediment adsorption of TBBPA were characterised using synchrotron radiation-Fourier transform infrared spectroscopy (SR-FTIRS). The results are displayed in Fig. 8. The sediment adsorbed with a small amount of TBBPA exhibited a weak displacement of the C–H stretching vibration from 2887–2896 cm^−1^ and 3500 cm^−1^ (O–H functional group), which indicate that TBBPA H-bonding force exists between TBBPA and the sediment. Furthermore, the stretching vibration peaks of the C = C bond on the benzene ring of TBBPA after being adsorbed were shifted to the right from the original (standard spectrum of TBBPA) at 1550 and 1472 to 1558 and 1505 cm^−1^, which indicates a strong π–π interaction force between the substrate sample and TBBPA.

## Conclusions

The adsorption kinetic results revealed that the adsorption of TBBPA by sediment was rapid in the first 2 h, and the adsorption amount reached 61.4% of the maximum adsorption amount at 2 h. Thus, it was a slow adsorption process, and the adsorption reached the apparent equilibrium after 48 h. The isothermal adsorption results revealed that both Langmuir and Freundlich models could well describe the isothermal process of TBBPA adsorption by sediment. The adsorption thermodynamic results revealed that the adsorption process of TBBPA by sediment was a spontaneous physical adsorption, which was a heat absorption reaction. Thus, the increase in the temperature was favourable to the adsorption process. The experiments of influencing factors revealed that the increase in the pH and ionic strength could inhibit the adsorption of TBBPA by sediment; the adsorption of TBBPA increased and then decreased with the increase in the HA concentration. The results of IR spectral characterisation revealed that a H-bonding and π-π force existed between sediment and TBBPA.

## Data Availability

All data generated or analysed during this study are included in this published article.
